# Augmenting turmeric, galingale, gotu kola extracts, and black sesame as prebiotics by probiotics

**DOI:** 10.14202/vetworld.2026.850-863

**Published:** 2026-02-28

**Authors:** Nipada Ranmeechai, Thapakorn Chumphon, Kanjana Pangjit, Saran Promsai

**Affiliations:** 1 Program of Bioproducts Science, Department of Science and Bioinnovation, Faculty of Liberal Arts and Science, Kasetsart University, Kamphaeng Saen Campus, Nakhon Pathom 73140, Thailand; 2 Major of Food Safety Innovation, Faculty of Agriculture at Kamphaeng Saen, Kasetsart University, Kamphaeng Saen Campus, Nakhon Pathom 73140, Thailand; 3 College of Medicine and Public Health, Ubon Ratchathani University, Ubon Ratchathani 34190, Thailand; 4 Division of Microbiology, Department of Science and Bioinnovation, Faculty of Liberal Arts and Science, Kasetsart University, Kamphaeng Saen Campus, Nakhon Pathom 73140, Thailand

**Keywords:** antioxidant activity, black sesame, functional food, gotu kola, prebiotic, probiotic, synbiotic fermentation, turmeric

## Abstract

**Background and Aim:**

Probiotic products are increasingly utilized in animal feed and the food industry to promote health benefits. Herbs such as turmeric (*Curcuma longa* L.), galingale (*Boesenbergia rotunda* (Linn.) Mansf.), gotu kola (*Centella asiatica* (L.) Urb.), and black sesame (*Sesamum indicum* L.) are rich in bioactive compounds with potential antioxidant, anti-inflammatory, and antimicrobial properties. However, their bioavailability can be limited, and fermentation with probiotics may enhance these attributes. This study investigated the formulation of a synbiotic product by fermenting aqueous extracts of turmeric, galingale, and gotu kola with multi-strain probiotics, supplemented with prebiotic black sesame extract, to improve probiotic viability and biofunctional activities, including enzyme inhibition and antioxidant effects.

**Materials and Methods:**

Seven probiotic strains (*Ligilactobacillus salivarius* KUKPS6202, *Lacticaseibacillus paracasei* KUKPS6201, *Lactobacillus acidophilus* KUKPS6107, *Lacticaseibacillus rhamnosus* KUKPS6007, *Limosilactobacillus reuteri* KUKPS6103, *Bacillus coagulans* KPS-TF02, and *Saccharomyces cerevisiae* subsp. *boulardii* KUKPS6005) were cultivated in a mung bean–soybean medium and used for fermentation of herbal extracts (10% w/v) at 37°C for 24 h. Black sesame extract (1% w/v) was added as a prebiotic. Antimicrobial activity was assessed via agar well diffusion against pathogens like *Aeromonas hydrophila* and *Bacillus cereus*. Probiotic viability was measured by plate counts. Bioactivities evaluated included α-amylase and α-glucosidase inhibition (IC50 values), lipase activity (using a commercial kit), and antioxidant potential via 2,2-diphenyl-1-picrylhydrazyl (DPPH) scavenging and ferric reducing antioxidant power (FRAP) assays. Data were analyzed using one-way analysis of variance and Tukey’s test (p < 0.05).

**Results:**

All probiotic strains grew effectively in the mung bean–soybean medium, with viable counts reaching 7-8 log CFU/mL. Fermented herbal extracts inhibited *A. hydrophila* and *B. cereus* (inhibition zones 7.33-9.00 mm) but showed limited activity against other pathogens. Black sesame extract significantly enhanced probiotic viability (p < 0.05) compared to unsupplemented extracts. Fermentation with *L. rhamnosus* yielded the lowest IC50 for α-amylase (5.96 mg/mL) and α-glucosidase (3.32 mg/mL). *L. reuteri* exhibited the highest lipase activity (384.77 mU/L after 24 h). Antioxidant activities were comparable across treatments in DPPH assay, but FRAP values were highest for *B. coagulans* (0.275 μg Trolox/mL), *S. boulardii* (0.264 μg Trolox/mL), and *L. salivarius* (0.252 μg Trolox/mL) (p < 0.05).

**Conclusion:**

Synbiotic fermentation with black sesame extract and multi-strain probiotics significantly boosts probiotic survival and biofunctional properties, offering potential as functional foods or animal feed supplements for metabolic health, antimicrobial protection, and antioxidant support. Future in vivo studies could validate these benefits.

## INTRODUCTION

Microbial fermentation represents one of the most efficient and economical approaches to food processing and preservation. Fermented foods are consumed globally and constitute an essential component of the human diet [1]. During fermentation, microbial enzymes degrade or transform undesirable constituents into beneficial products, thereby improving substrate quality through the formation of bioactive compounds [2]. This process alters food texture, flavor, aroma, and nutritional composition, generating distinctive sensory attributes along with associated health-promoting effects [3].

Probiotics, defined as beneficial microorganisms widely applied in the food industry and human nutrition, are commonly incorporated into fermented products and have been reported to exert diverse physiological benefits. These include modulation of intestinal microflora balance, enhancement of immune function, reduction of blood pressure and serum cholesterol levels, suppression of pathogenic intestinal bacteria, and antioxidant, antitumor, and antibacterial activities [4–6].

In Thailand, various medicinal herbs are traditionally integrated into food products to exploit their bioactive constituents. Several of these herbs, particularly those classified within the “Herbal Champion” group, hold significant commercial value for local farmers. Turmeric (*Curcuma longa* L.) is widely utilized as a natural yellow coloring agent, food additive, and spice and is well recognized for its pharmacological properties. These effects are mainly attributed to curcumin, which has been associated with anti-inflammatory activity and therapeutic potential against conditions such as Alzheimer’s disease, hypolipidemia, and mutagenicity [7]. Nevertheless, curcumin exhibits low bioavailability because of its lipophilic nature and poor aqueous solubility, leading to rapid hepatic metabolism and excretion following oral intake [8].

Galingale [*Boesenbergia rotunda* (Linn.) Mansf], locally referred to as krachai kaw in Thailand, has recently attracted attention as a potential prophylactic agent. Its rhizomes contain diverse bioactive compounds, including pinostrobin and panduratin A, which have demonstrated inhibitory activity against severe acute respiratory syndrome coronavirus 2 (SARS-CoV-2) infection [9–11]. Gotu kola [*Centella asiatica* (L.) Urb.], a commonly used medicinal herb, has shown a wide spectrum of beneficial effects, particularly in dermatological and neurological disorders. These therapeutic properties are largely attributed to triterpenoid glycosides such as madecassoside and asiaticoside, which possess anti-inflammatory, antioxidative, and anti-apoptotic activities [12, 13].

Black sesame (*Sesamum indicum* L.) seeds are rich in lipids and proteins and contain numerous bioactive compounds, including sesamin, sesamol, and episesamin. These constituents contribute to multiple health benefits, such as cholesterol regulation, improvement of blood lipid profiles, hepatoprotective and renoprotective effects, and antioxidant activity. In addition, black sesame seeds are abundant in dietary fiber, minerals, unsaturated fatty acids, vitamins, and various phytochemicals [14–16].

Diabetes mellitus is a metabolic disorder characterized by persistent hyperglycemia, commonly resulting from inadequate insulin secretion or impaired insulin action. A widely adopted therapeutic strategy involves the inhibition of carbohydrate-digesting enzymes, particularly α-glucosidase and α-amylase, to reduce postprandial blood glucose levels. Suppression of these enzymes delays carbohydrate digestion, decreases glucose absorption, and attenuates post-meal hyperglycemic peaks [17–19]. Likewise, inhibition of pancreatic lipase, the key enzyme responsible for dietary fat hydrolysis, has been proposed as an effective approach for obesity management by limiting fat absorption [20].

Although microbial fermentation has been extensively studied as a strategy to enhance the nutritional quality and functional properties of food substrates, most existing research has primarily focused on single plant materials or conventional probiotic products. There remains a clear lack of studies investigating the combined fermentation of medicinal herbs such as *C. longa*, *B. rotunda*, and *C. asiatica* together with nutrient-rich seeds such as *S. indicum*. Furthermore, although the individual bioactive compounds derived from these herbs and seeds have been reported to exhibit antioxidant, antimicrobial, antidiabetic, and anti-obesity activities, the effects of microbial fermentation on their biotransformation, stability, and enhanced biological efficacy have not been sufficiently elucidated. In particular, comprehensive evaluations of fermentation-induced modulation of key metabolic enzyme-inhibitory activities, including α-glucosidase, α-amylase, and pancreatic lipase, remain scarce. The mechanisms through which fermentation may improve the solubility, bioavailability, and functional performance of major phytochemicals such as curcumin, pinostrobin, panduratin A, madecassoside, asiaticoside, and sesamin are still poorly understood. As a result, scientific evidence supporting the formulation of fermented herbal-based functional foods targeting metabolic health is limited.

The present study aimed to investigate the impact of microbial fermentation on a composite substrate consisting of selected Thai medicinal herbs (*C. longa*, *B. rotunda*, and *C. asiatica*) combined with black sesame seeds (*S. indicum*). Specifically, this study sought to determine changes in bioactive compound composition and functional properties following fermentation, with particular emphasis on antioxidant activity, lipase production, and inhibitory effects against α-glucose and α-amylase. In addition, the study aimed to evaluate the potential of fermentation to enhance the bioavailability and health-promoting efficacy of key phytochemicals associated with the management of metabolic disorders. Ultimately, this research intended to provide a scientific basis for the development of innovative fermented functional food products with potential applications in the prevention and control of diabetes and obesity.

## MATERIALS AND METHODS

### Ethical approval and laboratory safety

All experiments were conducted *in vitro* and did not involve live animals or human participants; ethical approval was not required for this study. All laboratory procedures and data handling were followed. Institutional safety guidelines for the laboratory, according to Thailand’s Enhancing Safety Practices in Research Laboratories (ESPReL; number 2-0021-0052-7), were strictly observed.

### Study period and location

The study was conducted between 2021–2024 at the Microbiology Laboratory, Faculty of Liberal Arts and Science, Kasetsart University, Kamphaeng Saen Campus, Nakhon Pathom, Thailand.

### Raw materials

Thai ground herbs with particle sizes of approximately 0.3–0.5 mm, including turmeric (*C. longa*), galingale (*B. rotunda*), and gotu kola (*C. asiatica*), were purchased from Vejpongosot Yaowarat Co., Ltd., Bangkok, Thailand. The herbs were stored in a cold, dry chamber for approximately 2–3 days.

Black sesame seeds (*S. indicum*) were obtained from Thanya Farm Co., Ltd., Nonthaburi, Thailand. The seeds were stored in a cold, dry room for approximately 2–3 days and pulverized in a blender to obtain particle sizes of approximately 0.3–0.5 mm before being used in the studies.

### Preparation of probiotic strains

The probiotic strains used in this study were *Ligilactobacillus salivarius* KUKPS6202, *Lacticaseibacillus paracasei* KUKPS6201, *Lactobacillus acidophilus* KUKPS6107, *Lacticaseibacillus rhamnosus* KUKPS6007, *Limosilactobacillus reuteri* KUKPS6103, *Bacillus coagulans* KPS-TF02, and *Saccharomyces cerevisiae* subsp. *boulardii* KUKPS6005 [21, 22]. These strains were previously identified for their high probiotic potential and are listed by the Ministry of Public Health, Thailand. All probiotic strains were isolated from fermented foods purchased from different Thai local markets. All probiotic microorganisms were nonhaemolytic [21] and did not exhibit any antimicrobial resistance genes.

The probiotic bacteria were cultivated in nutrient broth (NB; Merck, Darmstadt, Germany) and de Man Rogosa and Sharpe medium (MRS; Merck, Darmstadt, Germany), while the probiotic yeast was cultured in yeast extract–malt extract medium (YM; Himedia, Mumbai, India). Incubation was performed at 37°C for 24–48 h. The cultures were stored at 4°C for short-term use and −20°C for long-term storage until further tests were needed.

### Probiotic cultivation in mung bean–soybean medium

A patented mung bean–soybean medium consisting of 1% (w/v) soybean, 1% (w/v) mung bean, and 0.5% (w/v) coconut sugar was prepared as previously described [23]. All probiotic strains were previously tested for growth enhancement. The samples were incubated at 37°C with shaking at 150 rpm to assess the effects of *L. salivarius*, *L. paracasei*, *L. acidophilus*, *L. rhamnosus*, *L. reuteri*, *B. coagulans*, and *S. boulardii* on growth in mung bean–soybean medium. Growth was monitored at 0, 24, 48, and 72 h using the spread plate count method.

### Preparation of aqueous herbal extracts fermented with multi-strain probiotics

Turmeric, galingale, and gotu kola were fermented with probiotic bacteria. Seven probiotic strains were cultivated in mung bean–soybean medium at 37°C for 48 h. The probiotic strain was separately mixed with 10% (w/v) dried herbal powder in water, heated to 100°C for 5 min, and cooled to 45°C.

A 25% (w/v) mixture of the probiotic inoculum (approximately 10^6^ colony-forming units [CFU]/mL) and herbal water (initial pH approximately 6) was added to the inoculum and incubated for 24 h at 37°C without agitation. The mixtures (final pH approximately 5) were placed in separate glass bottles and stored at 4°C until further use.

### Antibacterial activity assay

The antimicrobial activity of aqueous herbal extracts fermented with probiotics was tested using the agar well diffusion method [24] with some modifications. Pathogenic bacteria including *Aeromonas hydrophila*, *Bacillus cereus*, *Escherichia coli*, *Staphylococcus aureus*, *Staphylococcus epidermidis*, *Serratia marcescens*, *Salmonella Typhimurium*, *Proteus vulgaris*, and *Vibrio parahaemolyticus* were used as indicator strains.

Aqueous herbal extracts fermented with each single probiotic and multi-strain probiotics (100 μL) were added to the pathogen agar plates. The herbal extract was used as a negative control, while streptomycin at 0.1 mg/mL served as a positive control. The agar plates were incubated at 37°C for 48 h, and then the zones of inhibition were measured. The experiment was replicated three times. A clear zone around each well was used to assess the antibacterial activity.

### Prebiotic properties of black sesame extract in fermented aqueous herbal extracts

The aqueous herbal extract fermented with multi-strain probiotics was prepared as described above. A 1% (w/v) black sesame extract was added to the herbal extract before probiotic fermentation.

### Viable cell counts of probiotics in fermented extracts

The growth of probiotic strains in 1% black sesame extract in aqueous herbal extracts was compared with that in aqueous herbal extracts without sesame extract. Probiotic microorganisms were cultivated in 10 mL of MRS, NB, and YM broth media. The probiotics were then inoculated into each prebiotic-based medium and incubated for 24 h at 37°C.

Culture samples (1 mL) were collected after 0, 6, 12, 24, and 48 h. The growth of probiotics was measured using the dilution plate method [21] on different agar media. Briefly, 1 mL of sample was suspended in 9 mL of 0.85% normal saline before being diluted about 10 times until the suitable dilution was achieved. One hundred microliters of successive decimal dilutions were spread on medium agar (MRS, NA, or YM agar). The experiment was replicated four times. Then, the product fermented with black sesame extract in aqueous herbal extracts fermented with multi-strain probiotics was used for further analysis.

### α-Amylase inhibition assay

The fermented product was evaluated for the inhibition of α-amylase activity based on the modified methods of Kusano *et al*. [25] and Luyen *et al*. [26]. Potato starch (100 mg) was boiled in 5 mL of phosphate buffer (pH 7.0), cooled to room temperature, and combined with 50 μL of the fermented sample and 30 μL of 0.1 M phosphate buffer. After 5 min of pre-incubation, 20 μL of a 2 mg/mL α-amylase solution was added and incubated at 37°C for 15 min.

The reaction was stopped by adding 50 μL of 1 M HCl, followed by 50 μL of iodine solution. The absorbance was measured at 650 nm using a microplate reader (SPECTROstar Nano, BMG LABTECH, Ortenberg, Germany). Acarbose was used as the positive control. The half-maximal inhibitory concentration (IC50) was calculated. All determinations were performed at least three times.

The percentage inhibition (IC50) of absorbance at OD650 was plotted as a function of the acarbose concentration and was used to calculate the acarbose equivalent antioxidant capacity. The experiment was replicated three times.

### α-Glucosidase inhibition assay

The α-glucosidase enzyme inhibition assay, based on Luyen *et al*. [26] and Ali *et al*. [27], involved combining the fermented sample solution and 40 μL of 0.5 U/mL α-glucosidase in 120 μL of 0.1 M phosphate buffer (pH 7.0). After pre-incubation for 5 min, 40 μL of a 5 mM p-nitrophenyl-α-D-glucopyranoside solution was added, and the mixture was incubated for 30 min at 37°C.

The absorbance of the released p-nitrophenol was measured at 405 nm. Acarbose was used as the positive control. The IC50 was determined. All determinations were performed at least three times.

The percentage inhibition (IC50) of absorbance at OD405 was plotted as a function of acarbose concentration and was used to calculate the antioxidant capacity of acarbose. The experiment was replicated three times.

### Lipase activity measurement

Lipase activity was measured using a Lipase Assay Kit (Elabscience®, Houston, TX, USA) as described below. First, 10 μL of the control sample was added to the corresponding control well. Similarly, 10 μL of the fermented sample was added to each well. In the control well, 40 μL of the buffer solution was added, while in the sample wells, 40 μL of the substrate working solution was added.

The wells were thoroughly mixed for 5 s in a microplate reader (SPECTROstar Nano), followed by incubation at 37°C for 20 min. After incubation, 150 μL of the chromogenic working solution was added to each well, followed by thorough mixing for 5 s. Then, the wells were incubated in the dark for an additional 30 min at 37°C.

The optical density of each well was measured at 412 nm using a microplate reader. The experiment was replicated three times. The lipase activity was calculated using the following formula:

Lipase activity (mU/L) = (ΔA / ε × b) × f × T × 10^6^ × 1,000

where ΔA = ODsample − ODcontrol, ε = molar absorption coefficient (14,150 L/mol×cm), b = height of the reaction system (0.6 cm), T = incubation time of the reaction (20 min), and f = dilution factor of the sample before the test. One mol/L equals 10^6^ μmol/L.

The analytical sensitivity of the assay is 0.03 U/L. This was determined by adding two standard deviations to the mean optical density obtained when the zero standard was assayed 20 times, and the corresponding concentration was calculated (Elabscience®).

### Assessment of antioxidant activity

2,2-diphenyl-1-picrylhydrazyl (DPPH) radical scavenging activity assay

The antioxidant activity of the fermented samples was assessed using the 2,2-diphenyl-1-picrylhydrazyl radical scavenging assay (DPPH; Sigma-Aldrich, Saint Louis, MO, USA) following a modified protocol by Brand-Williams *et al*. [28]. Ten microliters of the sample supernatant was added to a test tube containing 190 μL of 0.06 mM DPPH solution in 95% ethanol. The mixture was vortexed thoroughly and incubated in the dark for 30 min at room temperature.

The absorbance was measured at 515 nm using a microplate reader (SPECTROstar Nano). The antioxidant activity was compared with that of a standard Trolox solution, with 95% ethanol as the blank. All determinations were performed at least three times.

The percentage inhibition (IC50) of absorbance at OD515 was plotted as a function of Trolox concentration and was used to calculate the equivalent antioxidant capacity. The experiment was replicated three times.

The DPPH radical scavenging activity (%) was calculated as [(Acontrol − Asample) / Acontrol] × 100, where Acontrol is the absorbance of the control (DPPH solution without the sample) and Asample is the absorbance of the sample after reaction with DPPH. Antioxidant activity was expressed as IC50 of Trolox divided by IC50 of the sample.

#### Ferric reducing antioxidant power (FRAP) assay

The FRAP assay was conducted according to Benzie and Strain [29] with slight modifications. The FRAP reagent consisted of 10 mM 2,4,6-tripyridyl-s-triazine, 20 mM FeCl_3_, and 300 mM acetate buffer (pH 3.6) mixed in a 10:1:1 ratio and incubated at 37°C before use.

Ten microliters of the fermented sample was mixed with 190 μL of FRAP reagent and incubated at room temperature for 30 min. The absorbance was measured at 593 nm using a microplate reader (SPECTROstar Nano). Trolox was used as the reference standard. The antioxidant capacity was expressed as IC50 values. All determinations were performed at least three times. The experiment was replicated three times.

### Statistical analysis

Experiments were conducted following a statistical design [30]. Data analysis was performed using the Statistical Package for the Social Sciences version 22 software (SPSS Inc., IBM®, Armonk, NY, USA). Tukey’s multiple comparisons test, one-way analysis of variance, and the independent-samples t-test were used to assess significant differences between groups. A significance level of p < 0.05 was considered statistically significant. Microsoft Excel was used for the IC50 and enzymatic calculations.

## RESULTS AND DISCUSSION

### Growth of probiotics in mung bean–soybean medium

The effects of mung bean and soybean-based medium on the growth of seven probiotic strains were evaluated after 72 h at 37°C. After a 24–72 h incubation period, all probiotic strains exhibited high growth in this medium. As shown in Table 1, the viable cell counts increased substantially compared to the initial time at 0 h. Based on these results, the mung bean–soybean medium provided a favorable environment for probiotic growth, as observed in another study on legume-based fermentation [31]. Notably, the lactic acid bacteria strains (*L. salivarius*, *L. paracasei*, *L. acidophilus*, *L. rhamnosus*, and *L. reuteri*) exhibited peak growth at 24 h, after which the viable cell count slightly declined at 72 h. This trend aligned with the findings of Ruiz de la Bastida *et al*. [32], where *L. paracasei* INIA P272, *L. rhamnosus* INIA P344, and *L. rhamnosus* GG reached their highest counts after 24 h of fermentation in soy beverages. Among the tested strains, *Bacillus coagulans* KPS-TF02 had the highest overall increase in viable count and maintained stable growth until 72 h, suggesting that *B. coagulans* is more resistant to environmental changes and can thrive during extended fermentation [33]. In addition, *Saccharomyces boulardii* KUKPS6005 exhibited steady growth but at a lower rate than the other bacterial strains. These findings confirm that mung bean and soybean are suitable substrates for probiotic fermentation, particularly for lactic acid bacteria and spore-forming *Bacillus* species. Future research should explore how different fermentation conditions (such as pH and temperature variations) influence probiotic survival and bioactive compound production [34]. Thus, the mung bean–soybean medium was used for the production of probiotic inoculum.

**Table 1:** Viable cell counts (log CFU/mL) of seven probiotics grown in mung bean–soybean medium and incubated at 37°C for 0, 24, 48, and 72 h.

Probiotic strain	0 h	24 h	48 h	72 h
*Ligilactobacillus salivarius* KUKPS6202	6.31 ± 0.017*	7.21 ± 0.017	7.30 ± 0.039	7.32 ± 0.028
*Lacticaseibacillus paracasei* KUKPS6201	6.24 ± 0.015	7.31 ± 0.008	7.28 ± 0.053	7.01 ± 0.033
*Lactobacillus acidophilus* KUKPS6107	6.24 ± 0.123	7.16 ± 0.077	7.10 ± 0.027	7.07 ± 0.115
*Lacticaseibacillus rhamnosus* KUKPS6007	6.12 ± 0.021	7.81 ± 0.285	7.34 ± 0.074	7.02 ± 0.065
*Limosilactobacillus reuteri* KUKPS6103	6.10 ± 0.072	7.30 ± 0.038	7.32 ± 0.055	7.22 ± 0.094
*Bacillus coagulans* KPS-TF02	4.11 ± 0.018	7.12 ± 0.016	7.78 ± 0.027	7.61 ± 0.045
*Saccharomyces boulardii* KUKPS6005	5.04 ± 0.019	6.30 ± 0.034	6.40 ± 0.059	6.37 ± 0.025

Values represent mean ± standard deviation of three independent experiments (n = 3).

### Antimicrobial activity

The antimicrobial activity of the fermented aqueous herbal extracts (turmeric, galingale, and gotu kola) using probiotic strains (*L. salivarius*, *L. paracasei*, *L. acidophilus*, *L. rhamnosus*, *L. reuteri*, *B. coagulans*, *S. boulardii*, and multi-strain) was compared against aqueous herbal extracts (negative control) and 0.01% streptomycin (positive control), as shown in Table 2 and Supplementary Figure S1 and S2. The antimicrobial activity against two major pathogens (*A. hydrophila* and *B. cereus*) was evaluated using the agar diffusion method. Based on the results, all the probiotics produced clear zones of inhibition, indicating their ability to suppress pathogen growth. The inhibition diameters of *A. hydrophila* (7.67–8.67 mm) and *B. cereus* (7.33–9.00 mm) suggested that the probiotic-fermented herbal extracts had moderate antimicrobial activity, likely due to the production of organic acids, hydrogen peroxide, and bacteriocins during fermentation [35]. With the other pathogens, the cells of *Vibrio parahaemolyticus* were destroyed, although no clear zone was formed (Supplementary Figure S1), indicating a different antimicrobial mechanism. The probiotic strains did not affect *E. coli*, *Proteus vulgaris*, *S. aureus*, *S. epidermidis*, *Serratia marcescens*, and *S. Typhimurium* (Supplementary data Figure S2). This may be due to the low concentration of active antimicrobial compounds or the high resistance of these bacteria. Due to the moderate inhibition activity, the minimum inhibitory concentration and minimum bactericidal concentration were not determined. However, other antibacterial activity tests, such as the co-inoculation test and adhesion activity, should be evaluated. Chumphon *et al*. [21] reported that several probiotic strains (*L. paracasei*, *L. acidophilus*, *L. reuteri*, *L. rhamnosus*, *L. salivarius*, *B. coagulans*, and *S. boulardii*) inhibited the growth of *B. cereus*, *E. coli*, *S. aureus*, *S. marcescens*, *S. Typhimurium*, *P. vulgaris*, *V. harveyi*, and *V. parahaemolyticus*. In addition, they reported that *L. rhamnosus* had strong antimicrobial activity against eight pathogens (*B. cereus*, *Pseudomonas aeruginosa*, *V. parahaemolyticus*, *S. marcescens*, *S. Typhimurium*, *A. hydrophila*, *Candida albicans*, and *E. coli*). In contrast, as reported elsewhere [36], *B. coagulans* inhibited any pathogens tested. The mechanism of probiotic antimicrobial activity was likely due to bacteriocins, which disrupt the bacterial cell wall, causing leakage of amino acids and inorganic salts and ultimately leading to pathogen inhibition [35]. Ensuring animal health and feed safety is a critical component of sustainable livestock and aquaculture production. Among the wide range of microbial hazards associated with animal production systems, *A. hydrophila* and *B. cereus* have gained increasing attention due to their ability to cause disease in animals and contaminate feed and feed ingredients. *A. hydrophila* exemplifies pathogens linked to aquatic environments and live animal disease, whereas *B. cereus* is a feed-borne hazard capable of surviving processing and producing harmful toxins. Together, they illustrate the interconnectedness of feed quality, environmental hygiene, and animal health. Safer feed formulations and alternative control strategies, such as probiotics, organic acids, and enhanced processing technologies. This aligns with global efforts to reduce AMR and promote sustainable animal production.

**Table 2:** Diameter of inhibition zones (mm) of intestinal pathogens by fermented herbal extracts with probiotics after 24 h at 37°C.

Treatment	AH	BC	EC	SA	SE	SM	ST	PV	VP
*Ligilactobacillus salivarius* KUKPS6202	7.67 ± 0.94^b^*	7.33 ± 0.47^b^	0.00 ± 0.00^b^	0.00 ± 0.00^b^	0.00 ± 0.00^b^	0.00 ± 0.00^b^	0.00 ± 0.00^b^	0.00 ± 0.00^b^	0.00 ± 0.00^b^
*Lacticaseibacillus paracasei* KUKPS6201	7.67 ± 0.94^b^	8.00 ± 0.00^b^	0.00 ± 0.00^b^	0.00 ± 0.00^b^	0.00 ± 0.00^b^	0.00 ± 0.00^b^	0.00 ± 0.00^b^	0.00 ± 0.00^b^	0.00 ± 0.00^b^
*Lactobacillus acidophilus* KUKPS6107	8.17 ± 0.62^b^	7.83 ± 0.62^b^	0.00 ± 0.00^b^	0.00 ± 0.00^b^	0.00 ± 0.00^b^	0.00 ± 0.00^b^	0.00 ± 0.00^b^	0.00 ± 0.00^b^	0.00 ± 0.00^b^
*Lacticaseibacillus rhamnosus* KUKPS6007	8.50 ± 0.41^b^	8.33 ± 0.47^b^	0.00 ± 0.00^b^	0.00 ± 0.00^b^	0.00 ± 0.00^b^	0.00 ± 0.00^b^	0.00 ± 0.00^b^	0.00 ± 0.00^b^	0.00 ± 0.00^b^
*Limosilactobacillus reuteri* KUKPS6103	8.00 ± 0.00^b^	7.83 ± 0.24^b^	0.00 ± 0.00^b^	0.00 ± 0.00^b^	0.00 ± 0.00^b^	0.00 ± 0.00^b^	0.00 ± 0.00^b^	0.00 ± 0.00^b^	0.00 ± 0.00^b^
*Bacillus coagulans* KPS-TF02	8.33 ± 0.47^b^	8.17 ± 0.24^b^	0.00 ± 0.00^b^	0.00 ± 0.00^b^	0.00 ± 0.00^b^	0.00 ± 0.00^b^	0.00 ± 0.00^b^	0.00 ± 0.00^b^	0.00 ± 0.00^b^
*Saccharomyces boulardii* KUKPS6005	8.17 ± 0.24^b^	8.67 ± 1.25^b^	0.00 ± 0.00^b^	0.00 ± 0.00^b^	0.00 ± 0.00^b^	0.00 ± 0.00^b^	0.00 ± 0.00^b^	0.00 ± 0.00^b^	0.00 ± 0.00^b^
Multi-strain probiotics	8.67 ± 0.47^b^	9.00 ± 0.82^b^	0.00 ± 0.00^b^	0.00 ± 0.00^b^	0.00 ± 0.00^b^	0.00 ± 0.00^b^	0.00 ± 0.00^b^	0.00 ± 0.00^b^	0.00 ± 0.00^b^
Aqueous herbal extract (negative control)	7.33 ± 0.47^b^	7.33 ± 0.47^b^	0.00 ± 0.00^b^	0.00 ± 0.00^b^	0.00 ± 0.00^b^	0.00 ± 0.00^b^	0.00 ± 0.00^b^	0.00 ± 0.00^b^	0.00 ± 0.00^b^
0.01% streptomycin (positive control)	22.00 ± 1.87^a^	21.33 ± 1.93^a^	18.67 ± 0.62^a^	30.17 ± 1.03^a^	18.83 ± 0.62^a^	8.50 ± 0.41^a^	14.67 ± 0.85^a^	8.83 ± 0.47^a^	22.50 ± 0.41^a^

Values represent mean ± standard deviation of three independent experiments (n = 3). Results with different lowercase superscripts within the same pathogen are significantly different based on Tukey’s multiple comparisons test (p < 0.05).

AH = *Aeromonas hydrophila*, BC = *Bacillus cereus*, EC = *Escherichia coli*, SA = *Staphylococcus aureus*, SE = *Staphylococcus epidermidis*, SM = *Serratia marcescens*, ST = *Salmonella Typhimurium*, PV = *Proteus vulgaris*, VP = *Vibrio parahaemolyticus*

### Viable cell counts of prebiotic black sesame extract in aqueous herbal extracts fermented with multi-strain probiotics

**Figure 1:**
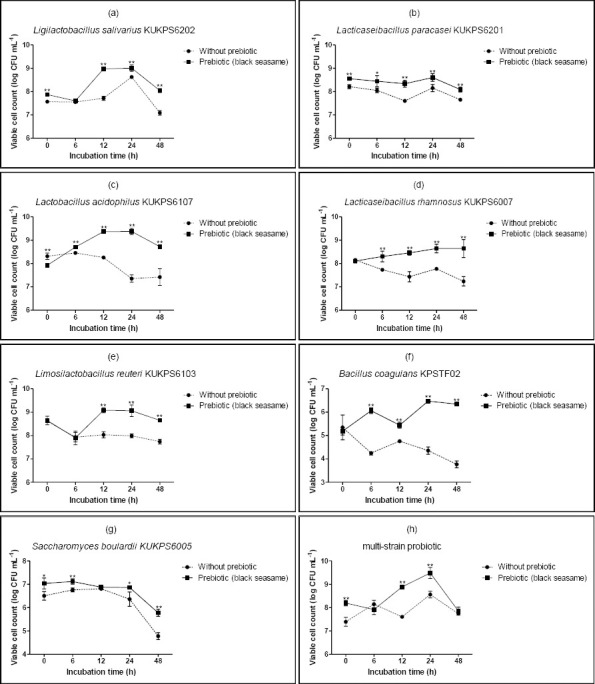
Viable cell counts of probiotic microorganisms: (a) *Ligilactobacillus salivarius* KUKPS6202, (b) *Lacticaseibacillus paracasei* KUKPS6201, (c) *Lactobacillus acidophilus* KUKPS6107, (d) *Lacticaseibacillus rhamnosus* KUKPS6007, (e) *Limosilactobacillus reuteri* KUKPS6103, (f) *Bacillus coagulans* KPS-TF02, (g) *Saccharomyces boulardii* KUKPS6005, and (h) multi-strain probiotics on: aqueous herbal extracts (without prebiotic) and aqueous herbal extracts mixed with black sesame extract (prebiotic). The error bar denotes the mean ± standard deviation (n = 4). Results with an asterisk for the same incubation time are significantly different based on an independent-sample t-test (p < 0.05).

Figure 1 shows the viable cell counts of *L. salivarius*, *L. paracasei*, *L. acidophilus*, *L. rhamnosus*, *L. reuteri*, *B. coagulans*, and *S. boulardii* during fermentation. The prebiotic potential of the black sesame extract was confirmed by the enhanced viable cell counts of all the probiotic strains tested. Some strains, especially *B. coagulans* and *L. salivarius*, had higher growth rates, indicating that they effectively digested the prebiotic ingredients. Black sesame extract had a substantial impact on probiotic development compared with aqueous herbal extracts alone (t-test; p < 0.05). Several mechanisms alone or in combination could be responsible for the observed increase in probiotic viability, with the potential growth enhancement pathways depending on nutrient availability. For example, the oligosaccharides and vital minerals found in black sesame extract provide the fermentable energy source for probiotic metabolic activity [37]. According to Wei *et al*. [16], black sesame fiber and polyphenols act as prebiotic substrates and specifically promote the growth of beneficial gut bacteria. Black sesame contains many antioxidants, including sesamin and sesamolin, which may shield probiotic cells from oxidative stress and increase their survival during fermentation [38]. The results of this study were corroborated by Wei *et al*. [16], who showed that prebiotics increased probiotic viability and metabolic activity. According to Fukuda *et al*. [38], black sesame components altered the gut microbiota composition and boosted probiotic survival in fermented foods. Our results were similar to those of Ruiz-Ojeda *et al*. [37], who reported that polyphenol-rich prebiotics greatly enhanced the growth of *Lactobacillus* and *Bifidobacterium* species. Overall, the results supported the notion that black sesame extract was a useful prebiotic that improved probiotic viability in aqueous herbal extracts. This prebiotic-probiotic combination may be beneficial in the creation of nutraceuticals and functional fermented foods that enhance gut health.

Notably, fundamental quality control parameters, such as total curcumin content and selected marker compounds, should be systematically evaluated to ensure reproducibility and improved characterization of raw materials. Bioactive compounds should be characterized using Fourier Transform Infrared Spectroscopy or GC-MS to enhance the development of commercial probiotic products.

### Enzyme inhibition

#### Alpha-amylase inhibition

Aqueous herbal extracts and black sesame extract fermented with multi-strain probiotics were evaluated for their ability to inhibit α-amylase after 0 and 24 h of incubation at 37°C. α-Amylase inhibition was moderately increased by fermentation, with *L. rhamnosus* showing the greatest inhibition after 24 h, with the lowest IC50 value. The multi-strain probiotics, *B. coagulans*, *L. salivarius*, *L. paracasei*, and *L. reuteri*, produced no notable alterations. With a greater IC50, the control group, which did not contain probiotics, showed less inhibition (Table 3). Probiotic fermentation did not appear to have a major effect on the structure of the α-amylase inhibitors, based on the mild effect of fermentation on α-amylase inhibition. Rather than targeting α-amylase, the fermented bioactive chemicals might preferentially target α-glucosidase. Although less effective, α-amylase inhibition is important for regulating starch digestion and may be used in conjunction with α-glucosidase inhibition to treat diabetes [39].

**Table 3:** Inhibition of α-amylase and α-glucosidase of herbal extracts with black sesame fermented with seven probiotics, multi-strain probiotics, and aqueous herbal extracts (negative control) after incubation for 0 and 24 h at 37°C.

Strain	α-Amylase inhibition IC50 (mg/mL) – 0 h	α-Amylase inhibition IC50 (mg/mL) – 24 h	α-Glucosidase inhibition IC50 (mg/mL) – 0 h	α-Glucosidase inhibition IC50 (mg/mL) – 24 h
*Ligilactobacillus salivarius* KUKPS6202	10.62 ± 0.87^abcA*^	9.05 ± 0.23^cdA^	16.70 ± 0.146^bcA*^	7.28 ± 1.034^bcB^
*Lacticaseibacillus paracasei* KUKPS6201	11.47 ± 1.72^abcA^	9.20 ± 0.21^bcdA^	15.64 ± 0.388^deA^	4.19 ± 0.264^deB^
*Lactobacillus acidophilus* KUKPS6107	13.04 ± 0.05^aA^	10.14 ± 0.58^abB^	16.03 ± 0.105^cdA^	6.20 ± 1.205^cB^
*Lacticaseibacillus rhamnosus* KUKPS6007	6.25 ± 0.13^dA^	5.96 ± 0.08^eA^	12.73 ± 0.358^gA^	3.32 ± 0.194^eB^
*Limosilactobacillus reuteri* KUKPS6103	9.22 ± 0.48^cA^	8.64 ± 0.09^dA^	16.02 ± 0.082^cdA^	8.68 ± 0.138^abB^
*Bacillus coagulans* KPS-TF02	9.75 ± 0.54^cA^	8.98 ± 0.23^cdA^	15.14 ± 0.261^efA^	6.68 ± 0.011^bcB^
*Saccharomyces boulardii* KUKPS6005	10.45 ± 0.48^bcA^	9.91 ± 0.23^abcA^	17.06 ± 0.010^bA^	10.14 ± 0.023^aB^
Multi-strain probiotics	11.20 ± 0.33^abcA^	10.44 ± 0.28^aA^	14.87 ± 0.178^fA^	5.42 ± 0.209^cdB^
Aqueous herbal extract (negative control)	12.31 ± 0.34^ab^	—	19.23 ± 0.079^a^	—
Acarbose (positive control)	0.65 ± 0.06^e^	—	0.39 ± 0.003^h^	—

* Values represent mean ± standard deviation of three independent experiments (n = 3). Results with different lowercase superscripts for the same incubation time are significantly different based on Tukey’s multiple comparisons test (p < 0.05). Results with different capital letters in the same row for each parameter are significantly different based on an independent-sample t-test (p < 0.05).

#### Alpha-glucosidase inhibition

After being incubated for 0 and 24 h at 37°C, the inhibition of α-glucosidase was evaluated in aqueous herbal extracts and black sesame extract fermented with multi-strain probiotics. As seen in Table 3, fermentation considerably increased enzyme inhibition, which may have anti-diabetic effects. *L. rhamnosus* showed the greatest α-glucosidase inhibition following fermentation, lowering the IC50 (p < 0.05). Additionally, IC50 values dropped significantly with multi-strain probiotics (p < 0.05). Enzyme-inhibitory activity was enhanced by fermentation, as demonstrated by the least inhibition being recorded in the non-fermented control. As a reference, the positive control (acarbose) showed the greatest inhibition. Fermented herbal extracts may slow down the digestion of carbohydrates and lessen postprandial glucose spikes, as shown by the notable decrease in the IC50 values. This improvement may have been caused by the bioactive substances, including phenolic acids, flavonoids, and peptides, that are generated during fermentation and block enzymes that break down carbohydrates [40]. Furthermore, probiotic metabolism might boost the synthesis of organic acids and short-chain fatty acids, which would further inhibit enzymes [41]. Among the studied probiotics, *L. rhamnosus* and the multi-strain probiotics had the highest inhibition levels, indicating their potential for use in functional foods that target blood sugar regulation. Based on these results, black sesame and fermented herbal extracts may help control blood sugar levels.

#### Enzyme inhibition

Lipase activity was measured in the black sesame extract and aqueous herbal extracts fermented with the multi-strain probiotics after 0 and 24 h of incubation at 37°C. Based on the results (Table 4), significant variations in activity were observed among the different probiotic strains. Some strains maintained high enzymatic activity after fermentation, whereas others declined.

**Table 4:** Lipase activity (mU/L) of herbal extracts with black sesame fermented with seven probiotics, multi-strain probiotics, and aqueous herbal extracts (negative control) after incubation at 37°C for 0 and 24 h.

Probiotic strain	0 h Incubation	24 h Incubation
*Ligilactobacillus salivarius* KUKPS6202	54.97 ± 21.68^eA^*	25.52 ± 15.46^bA^
*Lacticaseibacillus paracasei* KUKPS6201	190.42 ± 52.97^cdA^	11.78 ± 0.00^bB^
*Lactobacillus acidophilus* KUKPS6107	117.79 ± 17.34^deA^	11.78 ± 4.81^bB^
*Lacticaseibacillus rhamnosus* KUKPS6007	420.10 ± 64.93^bA^	21.59 ± 2.78^bB^
*Limosilactobacillus reuteri* KUKPS6103	881.43 ± 45.45^aA^	384.77 ± 59.74^aB^
*Bacillus coagulans* KPS-TF02	286.61 ± 23.72^cA^	56.93 ± 18.20^bB^
*Saccharomyces boulardii* KUKPS6005	51.04 ± 27.34^eA^	43.19 ± 24.68^bA^
Multi-strain probiotics	115.82 ± 43.10^deA^	56.93 ± 24.20^bA^
Aqueous herbal extract (negative control)	0.00 ± 0.00^e^	—

* Values represent mean±standard deviation of three independent experiments (n = 3). Results with different lowercase superscripts for the same incubation time are significantly different based on Tukey’s multiple comparisons test (p < 0.05). Results with different capital letters in the same row for each parameter are significantly different based on an independent-sample t-test (p < 0.05).

Among the tested strains, *L. reuteri* had the highest lipase activity, starting at 881.43 mU/L and decreasing to 384.77 mU/L after 24 h, which was still the highest among all strains. This suggests that *L. reuteri* plays a crucial role in lipid metabolism, potentially aiding fat digestion and absorption. These findings aligned with other studies indicating that *L. reuteri* produces high levels of bile salt hydrolase and lipopolysaccharide enzymes, which are essential for lipid metabolism [42]. Most of the tested probiotic strains significantly reduced lipase activity after fermentation. For example, *L. rhamnosus* showed a sharp decline (p < 0.05), whereas *B. coagulans* showed a decrease. These reductions suggested that lipase enzymes may have been consumed or degraded during fermentation. Conversely, *S. boulardii* and the multi-strain probiotics maintained a moderate lipase activity. Specifically, *S. boulardii* produced a slight decline, while the multi-strain probiotics produced a decrease, suggesting that combining multiple strains helped to stabilize enzymatic activity. No detectable lipase activity was observed in the control group (no probiotics), indicating that lipase activity originated from the metabolism of probiotics. The initial hydrolysis of lipids in the medium may have limited further enzymatic activity as fermentation progressed [43]. Some lipase are unstable during prolonged incubation, leading to decreased activity over time [44]. Additionally, microbial metabolism may shift from lipid breakdown to carbohydrate or phosphorus metabolism during fermentation, thereby reducing lipase production [45]. After 24 h, most probiotic strains experienced a substantial decline in lipase activity after 24 h, possibly due to substrate depletion or enzymatic instability. However, the multi-strain probiotic group retained a relatively stable activity level, suggesting that co-fermentation helped sustain enzymatic function over time. This stability may be attributed to synergistic interactions among probiotic strains, where some strains compensated for others’ enzymatic decline [46]. These findings support the use of multi-strain probiotics in functional food formulations targeting lipid metabolism. Their ability to maintain more stable lipase activity makes them suitable for applications in lipid metabolism supplements and fermented plant-based alternatives designed for weight management and metabolic health. Furthermore, this study reinforced previous research highlighting the advantages of multi-strain probiotics in enhancing metabolic stability and enzymatic function in functional foods [47].

### Antioxidant activities

Fermentation with multi-strain probiotics significantly enhanced the antioxidant activities of most strains, as measured by 2,2-diphenyl-1-picrylhydrazyl (DPPH) radical scavenging activity and FRAP assay. The antioxidant potential varied across strains, with *B. coagulans* showing the most substantial improvement in both assays (Table 5). The DPPH radical scavenging activity improved in most probiotic strains after fermentation. However, *B. coagulans* (p < 0.05) and *L. reuteri* (p < 0.05) showed the greatest increases in antioxidant activity, indicating strong antioxidant potential. Zhao *et al*. [48] reported that probiotics enhanced radical scavenging through the metabolism of polyphenols. In contrast, *L. paracasei* and *L. acidophilus* produced only moderate increases in DPPH activity, suggesting that some strains were less efficient in generating antioxidant metabolites during fermentation. In addition, *S. boulardii* and the multi-strain probiotics maintained similar values before and after fermentation, indicating a less pronounced effect on radical scavenging.

**Table 5:** Antioxidant activity (μg Trolox/mL sample) of herbal extracts with black sesame fermented with seven probiotics, multi-strain probiotics, and aqueous herbal extracts (negative control) using 2,2-diphenyl-1-picrylhydrazyl (DPPH) scavenging assay and ferric reducing antioxidant power assay incubation for 0 and 24 h at 37°C.

Treatment	DPPH scavenging 0 h	DPPH scavenging 24 h	FRAP 0 h	FRAP 24 h
*Ligilactobacillus salivarius* KUKPS6202	0.241 ± 0.0146^abA^*	0.251 ± 0.0058^aA^	0.281 ± 0.0097^abA^*	0.252 ± 0.0168^abcA^
*Lacticaseibacillus paracasei* KUKPS6201	0.211 ± 0.0063^cB^	0.233 ± 0.0069^aA^	0.258 ± 0.0097^abcA^	0.235 ± 0.0001^bcdB^
*Lactobacillus acidophilus* KUKPS6107	0.204 ± 0.0075^cB^	0.237 ± 0.0072^aA^	0.264 ± 0.0097^abcA^	0.235 ± 0.0168^bcdA^
*Lacticaseibacillus rhamnosus* KUKPS6007	0.216 ± 0.0037^bcB^	0.245 ± 0.0081^aA^	0.264 ± 0.0097^abcA^	0.219 ± 0.0001^dB^
*Limosilactobacillus reuteri* KUKPS6103	0.214 ± 0.0021^cB^	0.251 ± 0.0057^aA^	0.291 ± 0.0097^aA^	0.230 ± 0.0097^cdB^
*Bacillus coagulans* KPS-TF02	0.220 ± 0.0018^bcB^	0.260 ± 0.0091^aA^	0.230 ± 0.0349^bcA^	0.275 ± 0.0097^aA^
*Saccharomyces boulardii* KUKPS6005	0.263 ± 0.0111^aA^	0.241 ± 0.0100^aA^	0.230 ± 0.0257^bcA^	0.264 ± 0.0097^abA^
Multi-strains	0.260 ± 0.0024^aA^	0.244 ± 0.0084^aA^	0.224 ± 0.0256^cA^	0.241 ± 0.0097^bcdA^
No probiotic (control)	0.241 ± 0.0038^ab^	—	0.151 ± 0.000002^d^	—

* Values represent mean±standard deviation of three independent experiments (n = 3). Results with different lowercase superscripts for the same incubation time are significantly different based on Tukey’s multiple comparisons test (p < 0.05). Results with different capital letters in the same row for each parameter are significantly different based on an independent-sample t-test (p < 0.05).

The FRAP assay measures the ability of antioxidants to reduce Fe^3+^ to Fe^2+^ reflecting their overall redox stability and potential metal-chelating effects. Based on the current results, fermentation improved the FRAP values in some strains but reduced them in others. For example, *B. coagulans* (p < 0.05) demonstrated strong redox balance maintenance, whereas *S. boulardii* (p < 0.05) reduced oxidative stress. However, *L. rhamnosus* (p < 0.05) and *L. reuteri* (p < 0.05) were reduced, possibly due to metabolic shifts away from antioxidant compound production. *B. coagulans* and *S. boulardii* are ideal candidates for antioxidant-focused functional foods because of their superior antioxidant enhancement. *L. reuteri* and *B. coagulans* are known for their strong polyphenol metabolism, which may explain their high antioxidant enhancement [49]. *S. boulardii* produce extracellular metabolites that enhance antioxidant defense, contributing to high FRAP values. This variation could be attributed to differences in the metabolism of polyphenols and bioactive compounds in probiotic strains. Some probiotics produce extracellular metabolites that enhance antioxidant defense [50], while others degrade antioxidant compounds over time, leading to reduced FRAP activity.

The ability of probiotics to metabolize polyphenols varies by strain, influencing their antioxidant properties. Some probiotics break down complex antioxidant compounds into more bioavailable forms, thereby increasing antioxidant activity, whereas other strains may consume or degrade these bioactive compounds over extended fermentation periods, thereby reducing FRAP values. Probiotic strain selection and fermentation time optimization are crucial for maximizing antioxidant potential. The current results were consistent with other research in this respect, with Wang *et al*. [49] reporting that fermentation enhanced polyphenol bioavailability and antioxidant potential, particularly in *B. coagulans* and *S. boulardii*. Similarly, Hao *et al*. [50] noted that fermentation released bioactive peptides with antioxidant properties, consistent with the improved DPPH scavenging observed in the current study. However, some differences were observed compared with the results reported by Zhao *et al*. [51], in which FRAP values improved across all strains. In contrast, the current study identified certain strains with reduced FRAP values, suggesting that probiotic metabolism may shift during fermentation, affecting the stability of antioxidant metabolites. These findings highlight the strain-specific metabolic pathways influencing probiotic efficacy in enhancing antioxidant activity.

Antioxidant activity is fundamentally based on the suppression of free radical–mediated oxidation. Free radicals are highly reactive species containing unpaired electrons that are generated when covalent bonds are disrupted by external factors. They can induce cellular damage through oxidative stress. Phytochemicals mitigate these effects by interrupting free radical chain reactions, either through hydrogen atom donation or electron transfer to chelated metal ions.

In this study, fermentation of the aqueous herbal extract with probiotic strains significantly increased the levels of antioxidant compounds. Both the herbal extract and the probiotics enhanced the antioxidant capacity, likely through synergistic interactions that amplified the overall antioxidant activity. Further characterization of the bioactive compounds in the fermented products is required to fully elucidate the functional benefits of these probiotic formulations.

## CONCLUSION

This study demonstrated that synbiotic fermentation of aqueous extracts from *C. longa*, *B. rotunda*, and *C. asiatica* supplemented with prebiotic *S. indicum* extract significantly enhanced probiotic viability and biofunctional activities using seven strains: *L. salivarius* KUKPS6202, *L. paracasei* KUKPS6201, *L. acidophilus* KUKPS6107, *L. rhamnosus* KUKPS6007, *L. reuteri* KUKPS6103, *B. coagulans* KPS-TF02, and *S. cerevisiae* subsp. *boulardii* KUKPS6005. Key results included robust probiotic growth in mung bean–soybean medium (7-8 log CFU/mL), moderate antimicrobial activity against *A. hydrophila* and *B. cereus* (inhibition zones 7.33-9.00 mm), improved probiotic survival with black sesame (p < 0.05), superior α-amylase and α-glucosidase inhibition by *L. rhamnosus* (IC50 5.96 and 3.32 mg/mL, respectively), highest lipase activity with *L. reuteri* (384.77 mU/L), and enhanced antioxidant potential, particularly FRAP values for *B. coagulans* (0.275 μg Trolox/mL).

Practically, these findings suggest the synbiotic product could serve as a novel functional food or animal feed additive, promoting gut health, metabolic regulation, antimicrobial protection, and antioxidant support in livestock and human nutrition, aligning with sustainable agriculture in tropical regions like Thailand.

Strengths of the study include the integration of multi-strain probiotics for synergistic effects, utilization of locally sourced Thai herbs with high bioactive potential, and comprehensive evaluation of bioactivities, providing a strong foundation for eco-friendly product development.

However, limitations exist: the research was conducted *in vitro*, limiting insights into real-world bioavailability and efficacy; pathogen inhibition was selective and moderate; and results may vary with different herb batches or environmental conditions.

Future scope involves *in vivo* trials in animal models to validate health benefits, optimization of fermentation parameters for scalability, characterization of bioactive metabolites via advanced techniques like GC-MS, and exploration of commercialization for pet supplements or functional beverages.

In conclusion, this synbiotic fermentation approach harnesses natural resources to create value-added products with enhanced probiotic and biofunctional properties, offering promising avenues for health-promoting innovations in food and feed industries while supporting biodiversity and traditional knowledge.

## DATA AVAILABILITY

All generated data are included in the revised manuscript. Supplementary data and raw datasets are available from the corresponding author.

## AUTHORS’ CONTRIBUTIONS

NR: Software, methodology, investigation, formal analysis, data curation, validation, visualization, and writing–original draft. TC: Formal analysis and data curation. KP: Formal analysis, data curation, and writing–review and editing. SP: Supervision, resources, project administration, formal analysis, data curation, conceptualization, and drafted and revised the manuscript. All authors have read and approved the final version of the manuscript.
